# OOPS: Object-Oriented Polarization Software for analysis of fluorescence polarization microscopy images

**DOI:** 10.1371/journal.pcbi.1011723

**Published:** 2024-08-12

**Authors:** William F. Dean, Tomasz J. Nawara, Rose M. Albert, Alexa L. Mattheyses

**Affiliations:** Department of Cell, Developmental, and Integrative Biology, University of Alabama at Birmingham, Birmingham, Alabama, United States of America; Uppsala Universitet, SWEDEN

## Abstract

Most essential cellular functions are performed by proteins assembled into larger complexes. Fluorescence Polarization Microscopy (FPM) is a powerful technique that goes beyond traditional imaging methods by allowing researchers to measure not only the localization of proteins within cells, but also their orientation or alignment within complexes or cellular structures. FPM can be easily integrated into standard widefield microscopes with the addition of a polarization modulator. However, the extensive image processing and analysis required to interpret the data have limited its widespread adoption. To overcome these challenges and enhance accessibility, we introduce OOPS (Object-Oriented Polarization Software), a MATLAB package for object-based analysis of FPM data. By combining flexible image segmentation and novel object-based analyses with a high-throughput FPM processing pipeline, OOPS empowers researchers to simultaneously study molecular order and orientation in individual biological structures; conduct population assessments based on morphological features, intensity statistics, and FPM measurements; and create publication-quality visualizations, all within a user-friendly graphical interface. Here, we demonstrate the power and versatility of our approach by applying OOPS to punctate and filamentous structures.

## Introduction

Fluorescence microscopy has made it routine to characterize the subcellular localization and spatiotemporal dynamics of fluorescently labeled biomolecules in living cells. Unfortunately, conventional approaches are constrained by diffraction-limited resolution, meaning molecules within approximately 250 nm of one another cannot be distinguished [1]. Recent advances in super-resolution fluorescence microscopy have made it possible to circumvent the diffraction limit, offering greatly improved spatial resolution. However, while these approaches make it easier to understand where the molecules *are*, it is often equally desirable to understand how they are *organized*.

Studies of molecular architecture and orientation have traditionally relied upon electron microscopy (EM), which offers the highest spatial resolution among all imaging modalities. Still, the achievable resolution in single-molecule EM studies strongly depends on the rigidity of the sample, making it unsuitable for intrinsically flexible molecules. Additionally, the requirement for sample fixation abolishes all dynamic information and can disrupt native-state architecture. Moreover, EM is inherently non-specific, which makes it challenging to study the architecture and orientation of individual molecules, especially when they are embedded in the plasma membrane or integrated into larger subcellular complexes.

Fluorescence polarization microscopy (FPM) overcomes these challenges by exploiting the orientation-dependent (anisotropic) manner in which fluorophores absorb and emit light, making it possible to quantify the architecture of fluorescently labeled biomolecules in living cells [2,3]. In FPM, molecules of interest are rigidly labeled with fluorescent probes and then imaged while either modulating the orientation of the excitation polarization (excitation-resolved) [4–8], separating the emission into polarized components (emission-resolved) [3,9], or both [10,11]. By analyzing the polarization-dependent intensity changes, two key parameters can be retrieved: the orientational order, which reflects the relative alignment of the labeled molecules; and the orientation—hereafter referred to as the azimuth—which reflects the average direction along which the molecules are aligned. We focus here on the excitation-resolved modality, which can be configured on a standard widefield microscope by simply placing a polarization modulating device in the illumination path, making it widely accessible to cell biologists.

In widefield FPM, the order and azimuth measurements result from the combined contributions of all of the molecules in each diffraction-limited spot. In that sense, FPM provides sub-diffraction-scale structural information from diffraction-limited measurements, and can reveal the nanoscale architecture [4–16], orientational dynamics [17–20], conformational changes [21–23], and interactions [24–26] of fluorescently labeled biomolecules in their native cellular environments. Moreover, FPM excels in systems that remain challenging to study using more traditional structural approaches including membranes [27–41], membrane proteins [42, 43], cytoskeletal networks [44–54], and large multi-protein assemblies [4–12,14,16].

To fully realize the power of FPM, it is important to analyze individual biological structures, which can only be done with an object-based image analysis approach. For example, in an FPM study of the nuclear pore complex (NPC), Mattheyses et al. revealed the nanoscale organization of specific nucleoporin (nup) domains by comparing measured anisotropy patterns of individual NPCs to hypothesized models of NPC architecture [11]. Relatedly, Kampmann et al. combined anisotropy measurements with molecular modeling to elucidate the underlying organization of nups in the Y-shaped NPC subcomplex [10], which was later confirmed by cryo-EM [55]. A pair of FPM studies by Swaminathan et al. [56] and Nordenfelt et al. [9] showed that αVβ3 and αLβ2 integrins bound to immobilized ligands are collectively coaligned in focal adhesions by actin retrograde flow in migrating fibroblasts and lymphocytes, respectively. In both studies, FPM measurements of individual biological structures were used in combination with molecular modeling to reveal that the integrins were tilted with respect to the cell surface, supporting the cytoskeletal force hypothesis for integrin activation [9,56].

Despite the powerful advantages of FPM, its impact in cell biology has been hindered by the substantial image processing and mathematical analysis required to interpret the data. As a result, adoption of FPM has remained limited to a small number of specialized laboratories, often reliant on custom software solutions tailored to specific imaging setups or specimen geometries and frequently lacking comprehensive descriptions, thereby impeding their accessibility to the broader scientific community. Despite this need, very few open-source software tools have emerged that are capable of processing FPM data [57,58]. Moreover, while these tools fulfill their intended purposes, they nevertheless pose several limitations. In particular, due to the lack of automated object-based processing, it remains laborious and time-consuming to analyze individual biological structures. This is key, as object-based analysis enables quantification of biological diversity, spatial resolution of the subcellular distribution of order and orientation information, relation to underlying morphological features and intensity statistics, and measurement of azimuths relative to image features, all of which are required for interpreting FPM data in a biological context.

Here, we present OOPS (Object-Oriented Polarization Software), an advanced object-based image analysis software tailored for excitation-resolved FPM. By combining novel analyses with a high-throughput, object-based FPM image processing pipeline, OOPS empowers researchers to unlock the full potential of FPM. Importantly, in addition to facilitating object-based order measurements, OOPS provides the first fully automated method for calculating azimuth orientations relative to biological features of interest, which is vital for biological interpretation of the molecular orientation data. Additionally, OOPS offers flexible image segmentation; extensive object feature extraction; effortless object selection, filtering, labeling, and clustering; efficient and simultaneous processing of large datasets containing multiple groups of images; and highly customizable, publication-quality visualizations. To promote accessibility, all of the processing, analyses, and visualizations are housed within a convenient graphical user interface. We anticipate that OOPS will facilitate wider adoption of FPM, ultimately driving scientific discoveries.

### Design and implementation

OOPS is a GUI-based platform for object-based analysis of FPM images. The software is implemented in MATLAB and can be freely downloaded from GitHub. Usage requires MATLAB R2023b, as well as several MATLAB toolboxes, which are described in the software documentation.

### Expressions used to retrieve FPM statistics

Fluorescence Polarization Microscopy (FPM) aims to retrieve order and orientation statistics for a molecule or molecular complex. The core calculations are based on the anisotropic nature of fluorescence excitation with plane-polarized light. In a radiatively dominated regime, fluorescence intensity (*I*) is proportional to absorption probability such that:

$$I\propto {\left(\vec{E}\cdot \vec{\mu }\right)}^{2},$$

where $\vec{E}$ is a vector describing the amplitude and orientation of the excitation field and $\vec{\mu }$ is the absorption transition moment of the fluorophore [59]. For a plane-polarized excitation beam propagating along a fixed optical axis (z), $\vec{E}$ lies in the sample plane (x-y) and is described by its azimuthal angle (*ω*). The orientation of $\vec{\mu }$ is described by its polar (*θ*) and azimuthal (*φ*) angles. Here, the azimuthal angle is the angle between the positive x-axis and the orthogonal projection of $\vec{\mu }$ onto the x-y plane, while the polar angle is the angle between $\vec{\mu }$ and the z-axis. The fluorescence intensity measured using a particular excitation polarization (*I*_*ω*_) is:

$$I_{\omega }\propto {\left|\vec{E}\right|}^{2}{\left|\vec{\mu }\right|}^{2}\sin^{2}\left(\theta \right)\cos^{2}\left(\omega -\varphi \right)$$

Thus, for a single, static fluorophore, *I*_ω_ varies sinusoidally with *ω*, and the orientation of $\vec{\mu }$ can be determined by making multiple intensity measurements while rotating $\vec{E}$. For many fluorophores within a single diffraction-limited spot—assuming the rotational correlation time of the fluorophore is much slower than the fluorescence lifetime—the peak-to-peak amplitude of the sinusoid reflects the in-plane orientational order of the absorbing molecules, which we refer to interchangeably as “order” or *p*. The phase of the sinusoid gives the average azimuthal direction of the fluorophore transition dipole moments, which we refer to as “azimuth” or *α*. The order and azimuth can both be calculated using pixelwise image arithmetic from a series of images captured using excitation polarizations of 0°, 45°, 90°, and 135°:

$$S_{0}=(I_{0^{{}^{\circ}}}+I_{45^{{}^{\circ}}}+I_{90^{{}^{\circ}}}+I_{135^{{}^{\circ}}})/2$$

$$S_{1}=I_{0^{{}^{\circ}}}-I_{90^{{}^{\circ}}}$$

$$S_{2}=I_{45^{{}^{\circ}}}-I_{135^{{}^{\circ}}}$$

$$p=\frac{\sqrt{S_{1}^{2}+S_{2}^{2}}}{S_{0}}$$

$$\alpha =\frac{\mathrm{atan}2(S_{2},S_{1})}{2}$$

### Microscope setup

Excitation-resolved FPM data were collected on a Nikon-Ti 2 widefield microscope equipped with a custom excitation light path and a 60×, 1.49 NA oil-immersion objective. A continuous 488 nm laser (Coherent) was passed through a linear cleanup polarizer and an achromatic half waveplate before being focused on the objective back focal plane. The waveplate was placed in a motorized, rotating mount for electronic control of the excitation polarization. The emission was captured in four-image stacks on an ORCA-Flash 4.0 v3 sCMOS camera (Hamamatsu) using excitation polarizations of 0°, 45°, 90°, and 135°, which we refer to here as an “FPM stack”. Each excitation polarization refers to the in-plane orientation of the excitation electric field, which is measured anticlockwise with respect to the positive x-axis in the microscope coordinate system.

### Data requirements

OOPS uses BioFormats to import image data and can handle any open-source or proprietary microscopy image format supported by BioFormats [60], making it compatible with a wide array imaging systems. One of the major hurdles to broad adoption of FPM is that researchers employ disparate sets of nuanced correction and calibration procedures tailored to their specific microscope setups, acquisition settings, and downstream analyses. As a result, there is not a standard set of pre-processing operations, even when the excitation polarizations used to acquire the data are identical. For this reason, the only calibration procedure employed by OOPS is an optional, standard flat-field correction, using one or more flat-field calibration stacks captured using the same excitation polarizations as the FPM stack. However, the only required input is a single FPM stack, which can be corrected, calibrated, or otherwise pre-processed in any desired manner before it is imported.

While initially designed for excitation-resolved data, OOPS can easily be extended to emission-resolved setups, assuming the emission is split into 0°, 45°, 90°, and 135° components. In this case, or in the case of more nuanced acquisition setups, it may be desirable to change how the order statistic is calculated. To that end, we provide a configuration class (“CustomFPMStatistic.m”), with which users may programmatically define custom FPM outputs (User Manual). OOPS will dynamically add these properties to the data classes upon startup and seamlessly integrate each custom statistic into the interface.

### Data structure

**Fig 1:**
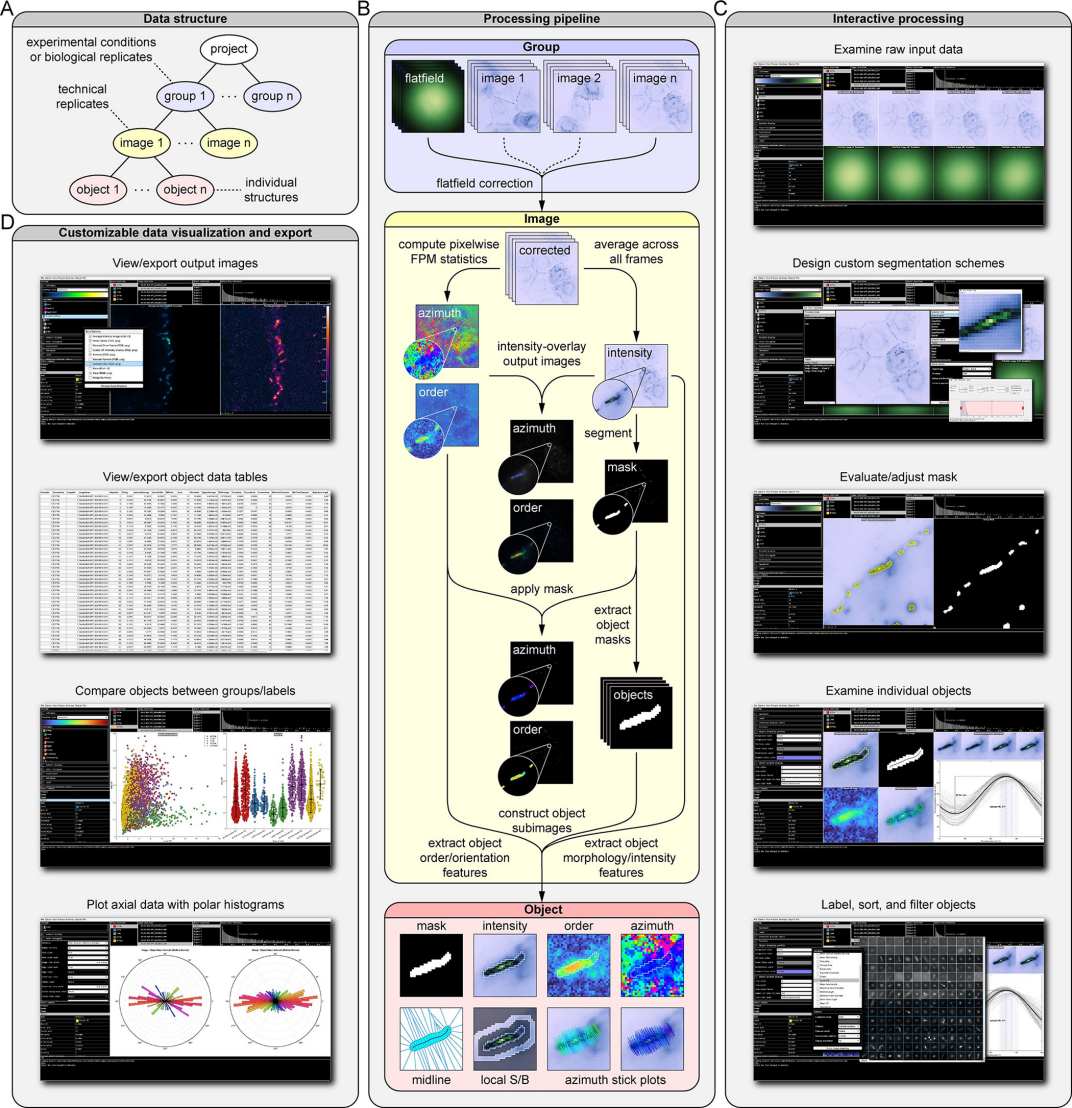
Overview of Object-Oriented Polarization Software (OOPS). (*A*) Hierarchical data structure used by the software. Projects are organized into “groups” representing biological replicates or experimental conditions, each containing a certain number of “images”. Each image contains “objects”, which store properties and statistics calculated for individual structures in the image. (*B*) Simplified processing pipeline for a single “image”, which includes flat-field correction, segmentation, calculation of FPM statistics, and object feature extraction. (*C*) Screenshots from the OOPS GUI showing examples of interactive data processing including examination of data, design of custom segmentation schemes, adjustment of image masks, and object manipulation. (*D*) Screenshots showing examples of the different image, plot, and data table visualizations available in the software.

To facilitate simultaneous analysis of datasets containing multiple experimental conditions or replicates, OOPS employs a hierarchical data structure (Fig 1A). Users create a “project” organized into “groups”, each corresponding to a different set of experimental conditions. Groups are composed of one or more “images”—each storing the raw FPM stack and any associated output data for a given technical replicate—along with one or more optional flat-field normalization stacks. Once the data have been loaded, processing results in the construction of “objects”, which contain all of the extracted features and output statistics for individual structures detected in an image (Fig 1B).

### Interactive data analysis, visualization, and export

Users interactively guide the data through each processing and analysis step with a flexible level of supervision (Fig 1C). Using default settings, users can quickly perform flat-field corrections, calculate pixelwise order and orientation statistics, segment images into objects, and extract object features for multiple images with only a few clicks. Alternatively, users can create custom segmentation schemes; adjust image masks; select, view, and label objects; and perform property-based object filtering, sorting, and clustering for more detailed analyses. Once the analyses are complete, users will visualize data with a wide variety of customizable plots and images (Fig 1D), which can be exported or copied directly from the software. Detailed object data tables can be exported for use in other plotting or statistics software. At any point, the project can be saved, closed, and then reopened at a later stage to continue the analysis.

### Image types and visualization options

The software enables users to export image data with a wide variety of customization options. To illustrate different types of output images, we fluorescently labeled the F-actin cytoskeleton of COS-7 cells with Alexa Fluor 488 (AF488) phalloidin—an ideal sample for FPM due to the fact that light absorption is strongly polarized along the direction parallel to the filaments [47] (S1 Fig). The FPM-specific image outputs are the pixelwise order and azimuth maps. These, along with the average intensity and mask images, can be directly exported for downstream analyses. In addition to the raw, grayscale outputs, the software enables the export of special output types that combine multiple statistics into a single image, each of which offer unique benefits (S1 Text and S1 Fig).

## Results

OOPS was designed to democratize FPM analysis for cell biology laboratories. Most subcellular molecular complexes appear as puncta or filaments when visualized by fluorescence microscopy, both of which are compatible with our software. We showcase this versatility through the analysis of two distinct datasets. First, we studied the domain-specific organization of desmocollin 2 (Dsc2) within individual desmosomes, punctate intercellular adhesion complexes. In addition, we demonstrate the importance of relative azimuth calculations, which provided key insights into the nanoscale geometry of Dsc2 ectodomains. Second, we applied OOPS to study the orientation of individual F-actin filaments labeled with AF488-phalloidin. Experiments in HUVECs grown in static conditions or under fluidic shear stress (FSS)—which induces a dramatic rearrangement of the actin cytoskeleton—show how azimuth measurements can be used to quantify changes in molecular orientation and reveal hidden relationships with underlying morphological features.

### Object-based FPM analysis reveals broad biological diversity in desmosomal cadherin architecture

Object-based analysis of order and orientation data obtained from FPM experiments is crucial for investigating biological variability, revealing subtle differences in architecture, and assessing the quality of the underlying data. To demonstrate this, we began by reanalyzing a previously published desmosome dataset [7]. Desmosomes are intercellular adhesive junctions responsible for maintaining mechanical integrity in epithelia and cardiac muscle [61]. Adhesion in desmosomes is conferred by a specialized class of transmembrane glycoproteins known as the desmosomal cadherins, desmogleins and desmocollins. The precise geometric arrangement of the cadherins—and in particular, their ectodomains—is predicted to be important for desmosome function [62] but has been challenging to study through conventional means, which have yielded conflicting results [63,64].

Here, we apply OOPS to study the organization of both the extracellular and intracellular domains of Dsc2, which is expressed in two alternatively spliced forms (“a” and “b”). The dataset comprises images of three unique Dsc2-EGFP chimeras which reveal the order and orientation of the Dsc2b ectodomain (ECTOb), Dsc2a ectodomain (ECTOa), and Dsc2a cytoplasmic domain (CYTO), along with a disordered control construct where EGFP has been attached to the Dsc2a C-terminus with a flexible linker (LINK). There are two ECTOa groups, one of similar quality to the other groups—ECTOa (good)—and a previously unpublished one of poor quality—ECTOa (poor)—which were collected in separate imaging sessions. ECTOa (poor), which suffers from low signal-to-noise ratios (SNRs), would typically be excluded from biological analysis but was retained here to illustrate key software features.

Desmosomes are close to the diffraction limit in size (~250 nm) and appear as either small puncta or slightly extended curvilinear structures in fluorescence microscopy. Therefore, images were segmented using the built-in “Puncta” scheme, which employs traditional morphological operations followed by threshold selection with Otsu’s method [65] (S1 Text). Individual connected components in the mask are used to construct objects, each representing an individual desmosome and associated with an extensive set of extracted features which include order and azimuth statistics derived from the polarization response data, morphological properties measured from the binary mask, and intensity information from the raw input data. Notably, after importing the image data into OOPS, all of the processing—including segmentation, computation of pixelwise order and azimuth images, and object feature extraction—only took ~40 s to perform for 83 images containing a total of 3,063 individual desmosomes.

To illustrate the additional information that can be gleaned from an object-based analysis, we began by plotting the average order ($\bar{p}$) in each image (image-order)—representing what would be obtained without OOPS—alongside that of the individual objects (object-order) for the ECTOb, CYTO, LINK, and ECTOa (good) datasets (S2 Fig). Both measures suggest the Dsc2 ectodomains are more highly ordered than the cytoplasmic tails; however, the image-based approach obscures the size and shape of the underlying distributions, making it difficult to assess biological variability. For example, while the image-order values are tightly grouped, the object-order values are highly variable, suggesting Dsc2 organization differs substantially between individual desmosomes. Moreover, the size and shape of the object-order distributions vary considerably between different constructs. Specifically, the ECTOa and ECTOb order distributions are much broader than those for the CYTO construct. This is a surprising finding that may have important implications for desmosome function. For example, order measurements of the Dsc2 ectodomains suggest that they have a highly ordered but flexible geometry, which might explain their ability to respond to a wide range of mechanical forces. Conversely, order measurements of the cytoplasmic tails suggest they are mostly disordered, which might be important for facilitating protein exchange during junctional remodeling. On the other hand, the narrower distribution of order values seen for the cytoplasmic tails suggests that they have a more consistent organization overall. One explanation for this is the fact that, while largely disorganized, the cytoplasmic tails are still partially constricted via their transmembrane attachments to the highly ordered ectodomains, allowing them to serve as a stable binding scaffold for intracellular adaptor and effector proteins.

**Fig 2:**
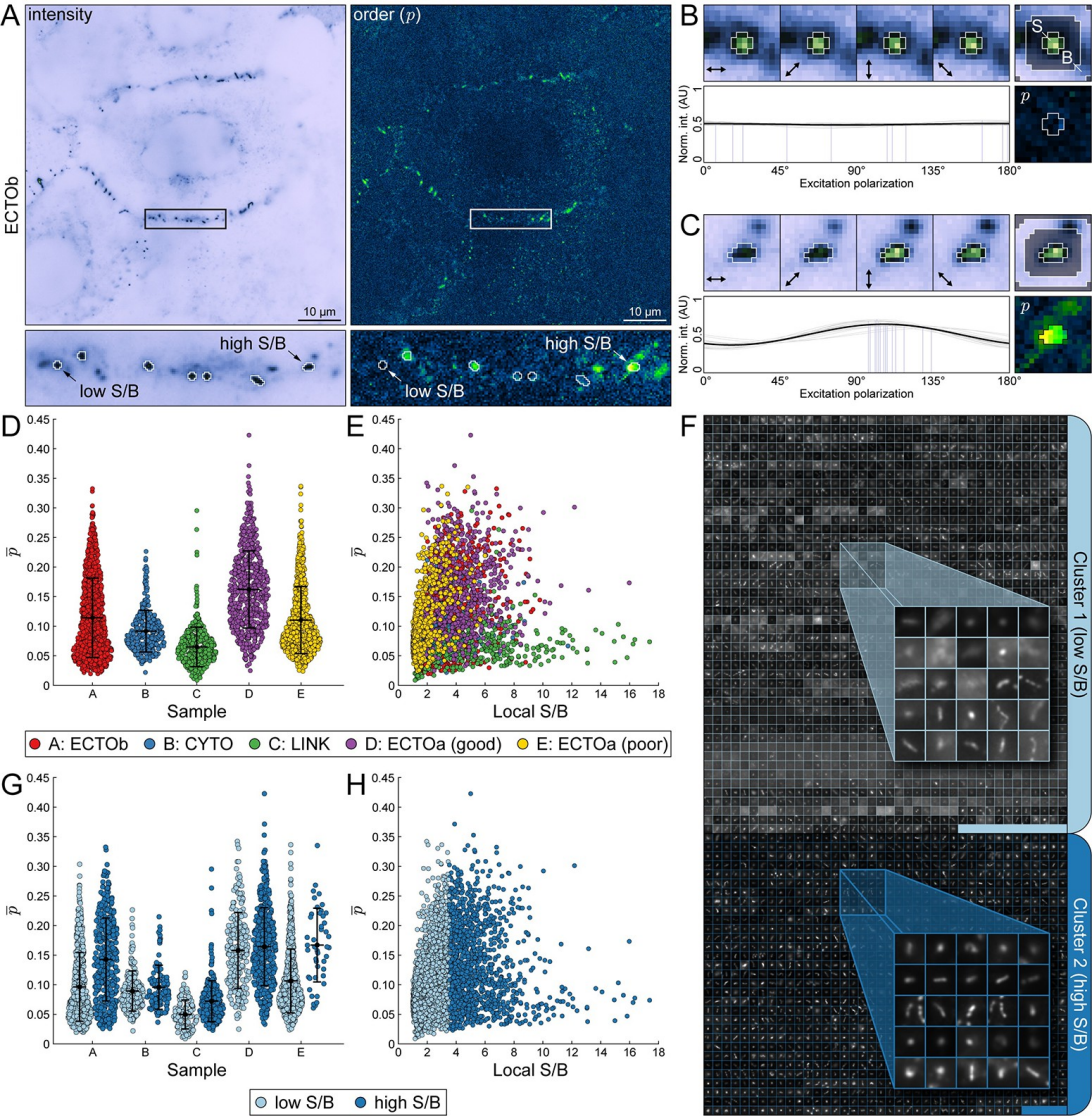
Object-based analysis reveals broad biological diversity in desmosomal cadherin architecture. Desmosomal cadherin order probes expressed in A-431 cells, imaged with FPM, and analyzed with OOPS to demonstrate object-oriented FPM image analysis. (*A*) Representative intensity (*top left*) and order (*p*, *top right*) images of the Desmocollin 2b extracellular order probe (ECTOb). A single cell-cell border containing several desmosomes is indicated with a rectangular ROI and magnified below. Objects detected after segmentation are enclosed by white boundaries. Two objects that differ in order and signal-to-background ratio (S/B) are indicated by arrows. (*B*) Closer inspection of the low S/B object in (*A*). (*Top left*) Object intensity images at each excitation polarization, normalized to the maximum intensity in the stack. Arrows indicate the direction of the excitation field. (*Top right*) Average intensity image, normalized to the maximum intensity. Labels indicate regions used to determine local S/B (*S*: signal; *B*: background). (*Bottom left*) Pixel intensity stacks normalized to the total intensity and fit to a generic sinusoid (*gray*: individual pixel fits; *blue*: individual pixel azimuths; *black*: average of all fits). (*Bottom right*) Object order image. Pixels used to calculate mean order are enclosed in a white boundary. (*C*) Same as (*B*), but for the high S/B object indicated in (*A*). (*D*) Swarm plots showing mean order ($\bar{p}$) for each object in the desmosome dataset, grouped by construct: ECTOb (*red*), CYTO (*blue*), LINK (*green*), ECTOa (good) (*purple*), ECTOa (poor) (*yellow*). (*E*) Scatter plot of $\bar{\rho }$ versus local S/B for each object, grouped as in (*D*). (*F*) All objects across all groups were sorted into low and high S/B clusters using k-means clustering. Objects are represented by their average intensity images, which are tiled and stitched together within each cluster: Cluster 1 (*light blue*; low S/B) and Cluster 2 (*dark blue*; high S/B). (*G*) Same as in (*D*) but grouped by both construct and cluster. (*H*) Same as in (*E*) but grouped by cluster.

An object-based analysis approach is also key for critical assessment of the quality of the underlying data. For example, one of the confounding factors that hinders precise and accurate determination of order and orientation parameters in FPM is the effect of noise on measurements of relative intensity changes [47]. A distinctive feature of our software is the ability to estimate the effect of this noise locally by calculating the local signal-to-background (S/B) ratio for each object. In a typical image, the local S/B can vary considerably [66], even between objects that are relatively close to one another (Fig 2A, *left*). In low S/B regions, polarization-dependent intensity changes are dominated by noise, effectively lowering the measured order (Fig 2A, *right*). For instance, when examining two objects from the same cell border—one with low S/B (Fig 2B) and one with high S/B (Fig 2C)—the latter appears more ordered, despite representing the same construct and being localized to the same cell border. Likewise, the objects in ECTOa (good) appear considerably more ordered than those in ECTOa (poor) (Fig 2D), which suffer from low S/B (Fig 2E).

To examine in-depth the effect of noise on measured order, we used k-means clustering to sort the objects into two groups, one with low S/B (Cluster 1) and one with high S/B (Cluster 2) (Fig 2F) (S1 Text). Replotting the data with objects grouped by cluster (Fig 2G and 2H) makes evident the effect of local S/B on observed order; in all cases, the mean order of the high S/B objects in Cluster 2 is greater than that seen in Cluster 1. Notably, when examining only objects in Cluster 2, the mean order of ECTOa (poor) is now remarkably close to that in ECTOa (good), despite differing considerably prior to clustering. Interestingly, while images in the ECTOb dataset did not appear visibly noisy, the mean order still increased substantially after filtering out objects with low S/B, highlighting the importance of considering *local* noise variations in what otherwise would have been assumed to be high quality data. This is especially important in FPM experiments, where even subtle differences in order can provide key biological insights. For example, the mean order of ECTOb following S/B filtering is less than that of ECTOa. The only difference between Dsc2a and Dsc2b is the truncation of the cytoplasmic plakoglobin-binding domain in the “b” form, suggesting Dsc2 ectodomain organization may be affected by intracellular interactions, as has been suggested previously [67] but never demonstrated.

### Relative azimuth calculations reveal nanoscale cadherin geometry in the desmosome

**Fig 3:**
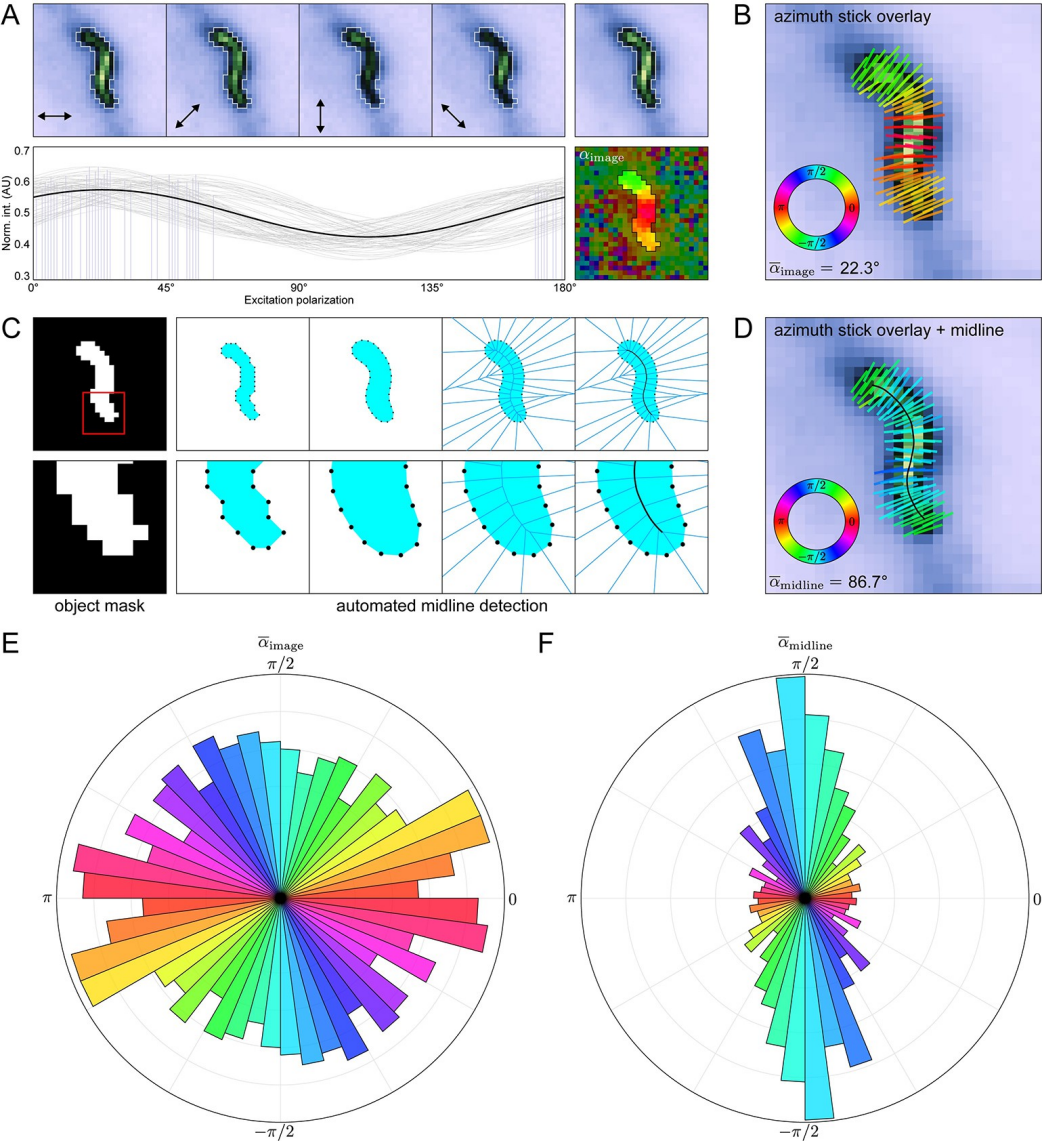
Relative azimuth calculations reveal nanoscale cadherin geometry in the desmosome. (*A*) Representative “S-shaped” object from the ECTOb dataset. (*Top left*) Object intensity images at each excitation polarization, normalized to the maximum intensity in the stack. Arrows indicate the direction of the excitation field. (*Top right*) Average intensity image, normalized to the maximum intensity. (*Bottom left*) Pixel intensity stacks normalized to the total intensity and fit to a generic sinusoid (*gray*: individual pixel fits; *blue*: individual pixel azimuths; *black*: average of all fits). (*Bottom right*) Object azimuth image. Pixel values represent the angle of the azimuths with respect to the excitation field in *I*_0°_ (*α*_image_). Background pixels are partially masked to highlight the object. (*B*) Average intensity image of the object in (*A*) with overlaid azimuth sticks, colored according to the direction, *α*_image_. (*C*) Simplified overview of the midline detection algorithm showing—from left to right—the binary mask defining the object; coordinates of the 8-connected perimeter pixels of the mask; boundary coordinates after dilation, smoothing, and linear arc interpolation; Voronoi diagram of the adjusted boundary points; and final midline detected from the central most edges of the Voronoi diagram after smoothing and interpolation. (*D*) Same as in (*B*), but with the midline overlaid and azimuth sticks colored according to their direction relative to the nearest midline tangent (*α*_midline_). (*E*) Polar histogram showing the distribution of ${\bar{\alpha }}_{image}$ for all objects in the ECTOb dataset. Object ${\bar{\alpha }}_{image}$ values initially in the range [−π/2,π/2] are duplicated and shifted by π to show each pair of equivalent, opposite directions. (F) Same as in (E), but for object ${\bar{\alpha }}_{midline}$ directions.

While order measurements yield important insights into how the labeled proteins are organized, they say little about the underlying geometry, which is better represented by the molecular azimuths. However, meaningful interpretation of these azimuths requires measuring from a suitable frame of reference—typically some biological feature of interest—which is unlikely to be aligned with the image itself. For eccentric, linear structures like F-actin filaments, this can be achieved by estimating the orientation of the azimuth sticks relative to the long axis of the object (S1 Fig). However, this procedure is cumbersome and time consuming, especially for datasets containing many discrete puncta. To illustrate this challenge, we consider a representative desmosome with an “S-shaped” morphology from the ECTOb dataset (Fig 3A, *top*). For each pixel in the object, the azimuth is simply the phase of a sinusoid fit to its polarization-dependent intensity profile (Fig 3A, *bottom*). In this context, the azimuth is an angle measured with respect to the direction of the excitation field in *I*_0°_ and therefore represents the average in-plane direction of the fluorescent dipoles relative to the horizontal direction in the image (*α*_image_). When the azimuths are plotted as sticks overlaid upon the average intensity image, they appear to be oriented perpendicular to the junction, yet this behavior is not reflected by the mean azimuth (${\bar{\alpha }}_{image}$ = 22.3°) (Fig 3B) (S1 Text).

Indeed, the raw azimuth values measured with respect to the microscope coordinate system are not biologically informative. To facilitate quantification of azimuth orientations with respect to the objects themselves, we developed an automated midline detection algorithm (Fig 3C) (S1 Text). In brief, we start with a set of points representing the 8-connected boundary of the object mask which is dilated, smoothed, and then used to construct a Voronoi diagram, the central most edges of which comprise the object midline. The midline points are extracted and smoothed, and the local orientation of the object is estimated by assigning to each pixel the value of the nearest midline tangent. Finally, azimuths are recalculated with respect to the orientation of the midline (*α*_midline_) using circular statistics (S1 Text). For the S-shaped desmosome, the mean azimuth measured with respect to the midline more accurately reflects the qualitative appearance (${\bar{\alpha }}_{midline}$ = 86.7°) (Fig 3D).

To demonstrate the significance of this approach, we compared azimuth calculation methods for all objects in the ECTOb dataset. When not adjusted to account for the orientation of the objects, azimuths appear randomly distributed and provide no information about the underlying molecular geometry (Fig 3E). On the other hand, when measured with respect to the midlines, azimuths are overwhelmingly perpendicular to the objects (Fig 3F). This indicates a consistent organization of the labeled molecules with respect to their larger complexes, while also suggesting that individual Dsc2 ectodomains are arranged symmetrically about the membrane normal. Importantly, this information would be entirely lost if azimuths were not adjusted to account for the local orientation of the objects, highlighting the power and utility of our approach. To the best of our knowledge, this is the first and only completely automated method that enables the determination of azimuth orientations relative to individual image features.

### Quantifying cytoskeletal reorganization in cells exposed to fluidic shear stress

**Fig 4:**
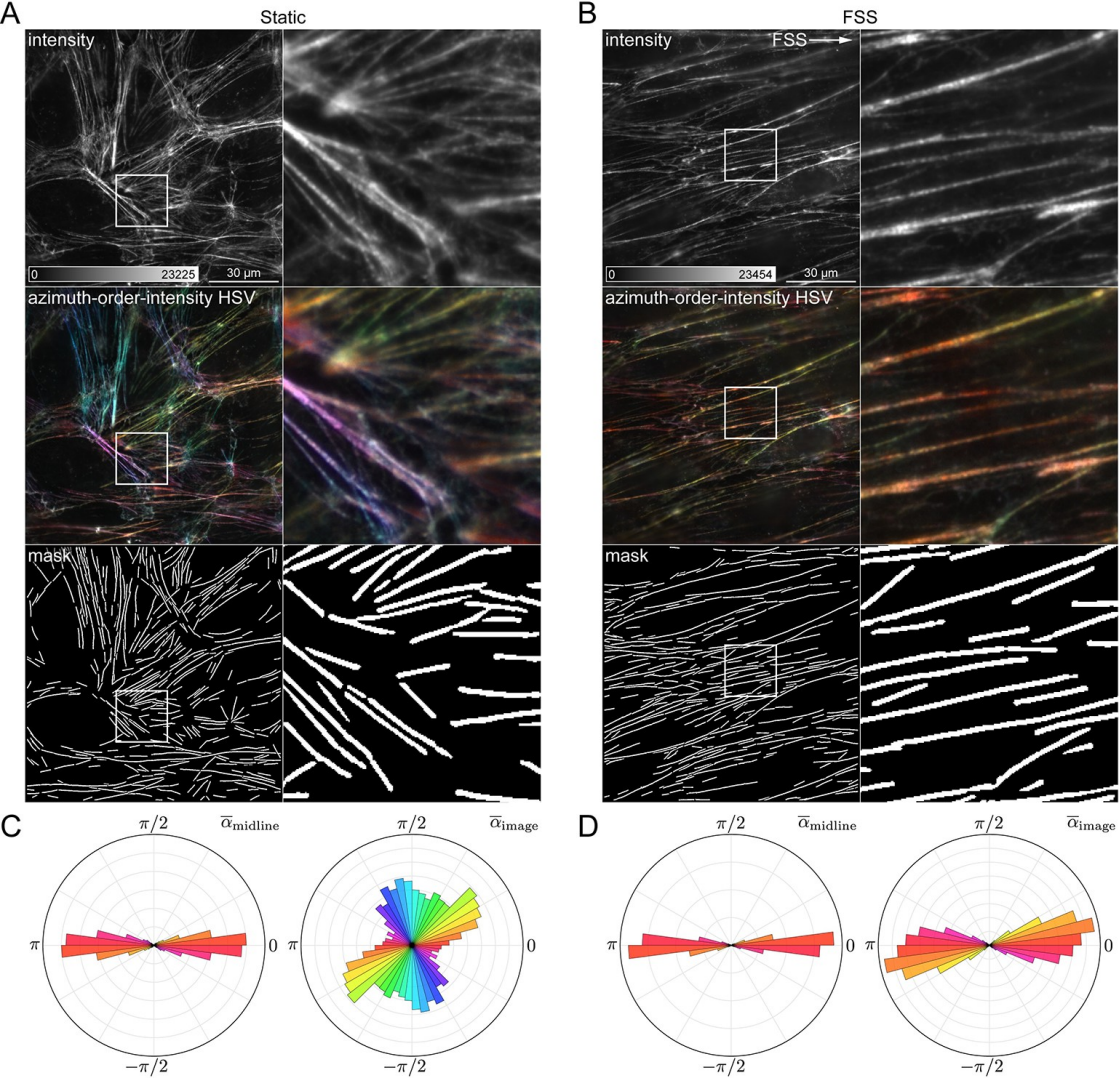
Quantifying cytoskeletal reorganization in cells grown under flow. (*A*) Human umbilical vein endothelial cells (HUVECs) grown in static conditions, labelled with AF488-phalloidin, and imaged with FPM. From top to bottom: average intensity image, azimuth-order-intensity HSV image, and binary mask showing locations of detected filaments. The region indicated by a square ROI is shown magnified to the right. (*B*) Same as (*A*) but for HUVECs grown under fluidic shear stress (FSS). White arrow indicates the flow direction. (*C*) Polar histograms showing ${\bar{\alpha }}_{midline}$ (*left*) and ${\bar{\alpha }}_{image}$ (*right*) distributions for filaments in cells grown statically. (*D*) Same as (*C*), but for cells grown under FSS.

The object-oriented analysis approach is fully compatible with any sample morphology, given an appropriate mask. Here, to demonstrate the flexibility of the approach, we used OOPS to analyze the orientation of the F-actin cytoskeleton in HUVECs grown either in static conditions or under fluidic shear stress (FSS). In cells grown under static conditions, the filaments appear randomly oriented (Fig 4A), whereas those in the cells grown under FSS appear to be preferentially oriented along the direction of the flow (Fig 4B). For both sets of samples, images were segmented using the built-in “Filaments” scheme, designed to detect linear, extended structures (S1 Text). In both groups, the azimuths are oriented along the direction of the filaments and therefore report the directions of the filaments themselves (Fig 4C and 4D). For cells grown statically, azimuths are randomly oriented with respect to the image, consistent with the appearance of the filaments. On the other hand, for cells grown under FSS, the azimuths are roughly aligned with the horizontal direction in the image, consistent with a reorganization of the actin cytoskeleton.

**Fig 5:**
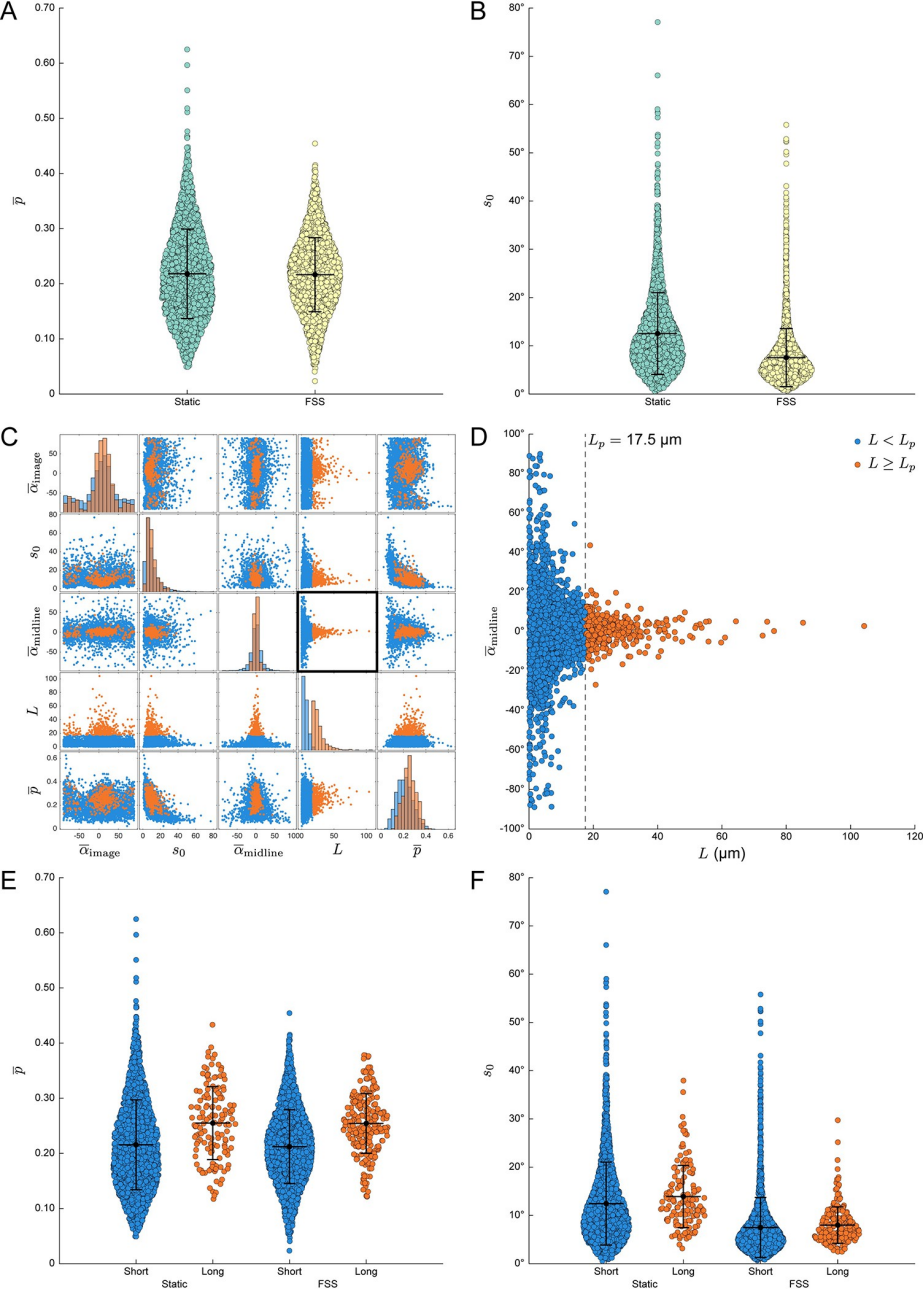
Object-based analysis reveals hidden relationships between F-actin filament architecture and morphology. (*A*) Swarm plots showing mean order ($\bar{p}$) for each segmented filament in cells grown in static conditions (*green*) or under fluidic shear stress (FSS, *yellow*). (*B*) Swarm plots showing azimuth circular standard deviation (*s*_0_) for the same groups in (*A*). (*C*) Scatter plot matrix exported from OOPS showing relationship between filament length (*L*) and FPM order and azimuth statistics for all filaments across both growth conditions, sorted into two groups: “Short” (*blue*, *L* < 17.5 μm) and “Long” (*orange*, *L* ≥ 17.5 μm). (*D*) Magnified version of the scatterplot highlighted by a black square in (*C*), showing the relationship between *L* and ${\bar{\alpha }}_{midline}$. A dashed black line denotes the cutoff point between “Short” and “Long” filaments, which was chosen to approximate the reported persistence length (*L*_*p*_) of phalloidin-stabilized F-actin, *L*_*p*_ = 17.5 μm. (*E–F*) Same as in (*A–B*) but grouped based on growth condition and filament length.

We hypothesized that actin filaments in cells exposed to FSS might display increased order due to mechanical strain imposed by the flow. Instead, we found the mean order to be virtually identical across both conditions (Fig 5A). This suggests that while FSS induces a dramatic change in actin organization at a cellular level, the nanoscale architecture is preserved. However, it remains unclear whether the underlying architecture is consistent along the length of each filament. To address this, we quantified the azimuthal disorder, defined here as the circular standard deviation (*s*_0_) of the azimuths in each filament (S1 Text). While order measurements reflect the mutual alignment of AF488 dipoles in each pixel, the azimuthal disorder is inversely proportional to the coherence of the individual pixel azimuths in each filament. Surprisingly, filaments in cells exposed to FSS displayed lower azimuthal disorder compared to those grown under static conditions, despite being similarly ordered (Fig 5B).

To better understand this discrepancy, we next sought to determine the impact of filament morphology on the observed FPM statistics. Among all of the calculated morphological features, the order and orientation statistics seemed to correlate most strongly with the length of the individual filaments (Fig 5C). For example, there is a clear relationship between filament length (*L*) and relative azimuth orientation (*α*_midline_), with only the longer filaments displaying azimuths consistently oriented parallel to their long axes (Fig 5D). Interestingly, there is a noticeable inflection point in the plot of *α*_midline_ versus *L* which is consistent with reported values [68,69] of the persistence length (*L*_*p*_) of phalloidin-stabilized F-actin (*L*_*p*_ = 17–18 μm). This led us to wonder whether filaments longer or shorter than the persistence length would be ordered differently. To that end, we used OOPS to resort the data into two groups, labeling each filament as either “short” (*L*<*L*_*p*_) or “long” (*L*≥*L*_*p*_), with *L*_*p*_ = 17.5 μm. Indeed, we found that the longer filaments were considerably more ordered than the shorter filaments (Fig 5E). Importantly, this increase was consistent across both growth conditions, suggesting it is related to the underlying morphology of the filaments.

The increased order seen in filaments longer than the characteristic persistence length is likely a result of increased flexural rigidity, although other factors might also play a role. For example, one possibility is that the shorter filaments simply have a higher ratio of ends to length; the greater contribution of the highly dynamic ends in the shorter filaments might lead to decreased order compared to their longer counterparts, which are predominantly composed of the more stable central regions with relatively minimal contributions from the ends. Alternatively, it is possible that the longer filaments represent higher order bundles, which could also increase the measured order. However—in contrast to the order measurements—we found that the azimuthal disorder was largely independent of the underlying morphology (Fig 5F). FSS apparently increases the collective alignment of pixel azimuths in each filament irrespective of filament length and without an accompanying change in order. This might be due to the fact that filaments in cells grown under FSS adopt more linear, extended conformations with fewer branchpoints, which could lead to more well-aligned azimuths along the length of each filament. Together, these data demonstrate how an object-based FPM analysis approach can reveal interesting, otherwise hidden relationships between nanoscale architecture and underlying morphological features that are often ignored in FPM studies.

## Conclusions

This study introduces OOPS as a flexible, GUI-driven MATLAB package for object-based analysis of molecular order and orientation in FPM images. It provides an intuitive platform for object-based image analysis, enabling users to uncover meaningful, otherwise hidden features in the underlying data. OOPS is broadly compatible with common sample geometries and labeling strategies, as demonstrated here via the analysis of punctate structures labeled with fluorescent proteins and filamentous structures labeled with small molecule fluorophores.

## Supporting information

S1 Fig Image types and visualization options. Filamentous actin (F-actin) in COS-7 cells labelled with AF488-phalloidin and imaged with FPM to illustrate different output image types. (*A*) Order-intensity overlay (*middle*), made by combining the order (*lower right*) and intensity (*upper left*) images, with the latter acting as an opacity mask. (*B*) Azimuth-order-intensity HSV (*middle*), made by combining the azimuth (*lower right*), order (*A*), and intensity (*A*) images, which are used to set the hue, saturation, and value, respectively. (*C*) Magnified images of individual filaments indicated by the square ROIs in (*A*) and (*B*) showing—from left to right—the intensity, order-intensity overlay, azimuth-order-intensity HSV, and azimuth stick overlay. A small segment of each filament is highlighted with a square ROI and shown as a magnified inset to illustrate the expected alignment of the azimuths with the long axis of the filament. See S1 Text for more details.
(TIF)

S2 FigComparison of image- and object-based order measurements.Swarmplots of mean order ($\bar{p}$) for all of the images and the objects they contain for the ECTOb, CYTO, LINK, and ECTOa (good) datasets. Values correspond either to the average order among all masked pixels in an image (*blue*) or the average order among all of the pixels defining each object (*red*).(TIF)

S1 Data Supplemental data. Excel spreadsheet containing underlying numerical data, object labels, and source image names for Figs 2D, 2E, 2G, 2H, 3E, 3F, 4C, 4D, 5A, 5B, 5C, 5D, 5E, 5F and S2.
(XLSX)

S1 Text Supplemental methods. (PDF)
