# Supplementary figures and images for: OOPS: Object-Oriented Polarization Software for analysis of fluorescence polarization microscopy images
Source: PLoS Comput Biol. 2024 Aug 12;20(8):e1011723. doi: 10.1371/journal.pcbi.1011723 (PMC11341096; doi:10.1371/journal.pcbi.1011723)

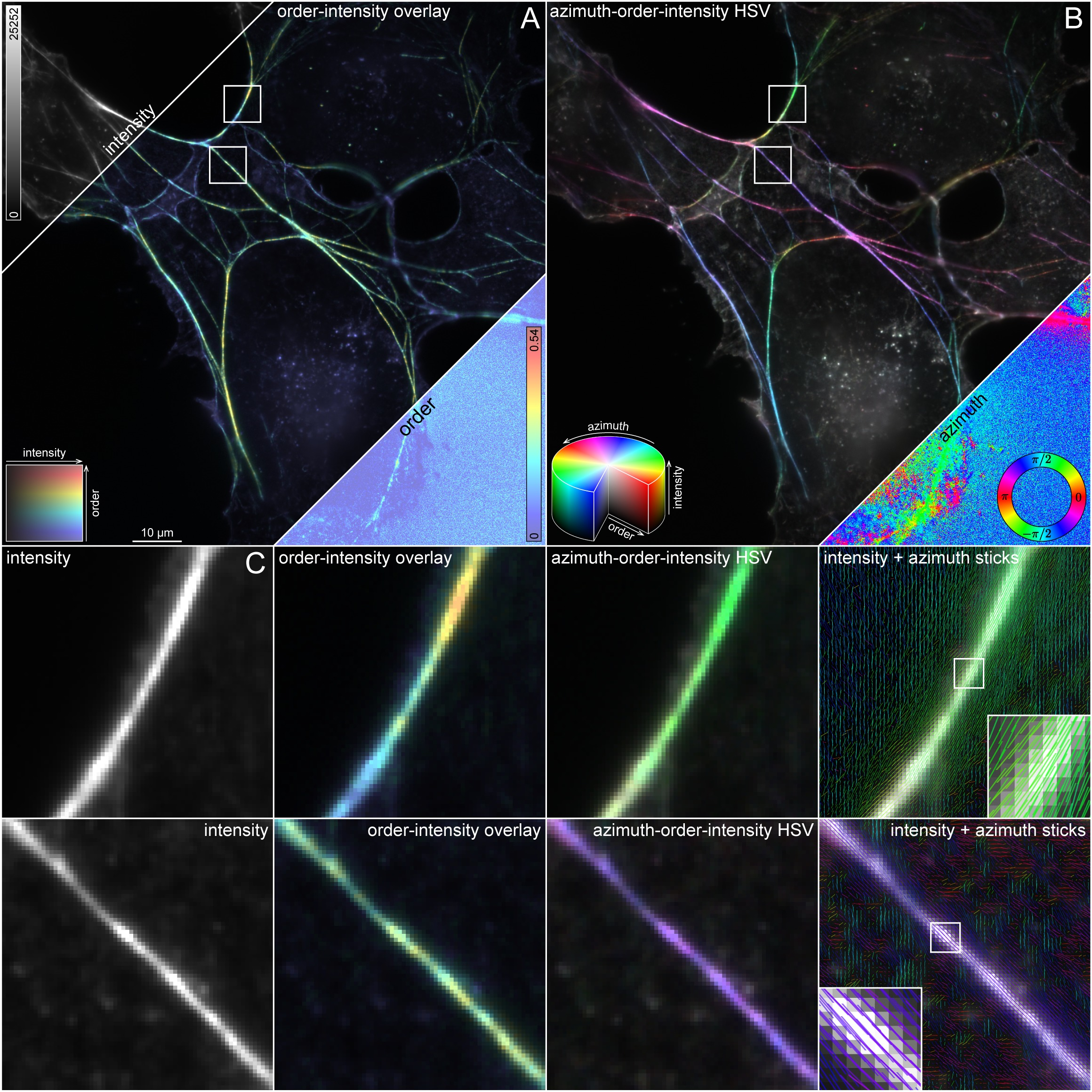

Supplement: S1 Fig — Filamentous actin (F-actin) in COS-7 cells labelled with AF488-phalloidin and imaged with FPM to illustrate different output image types. (A) Order-intensity overlay (middle), made by combining the order (lower right) and intensity (upper left) images, with the latter acting as an opacity mask. (B) Azimuth-order-intensity HSV (middle), made by combining the azimuth (lower right), order (A), and intensity (A) images, which are used to set the hue, saturation, and value, respectively. (C) Magnified images of individual filaments indicated by the square ROIs in (A) and (B) showing—from left to right—the intensity, order-intensity overlay, azimuth-order-intensity HSV, and azimuth stick overlay. A small segment of each filament is highlighted with a square ROI and shown as a magnified inset to illustrate the expected alignment of the azimuths with the long axis of the filament. See S1 Text for more details. (TIF) [file pcbi.1011723.s001.tif]

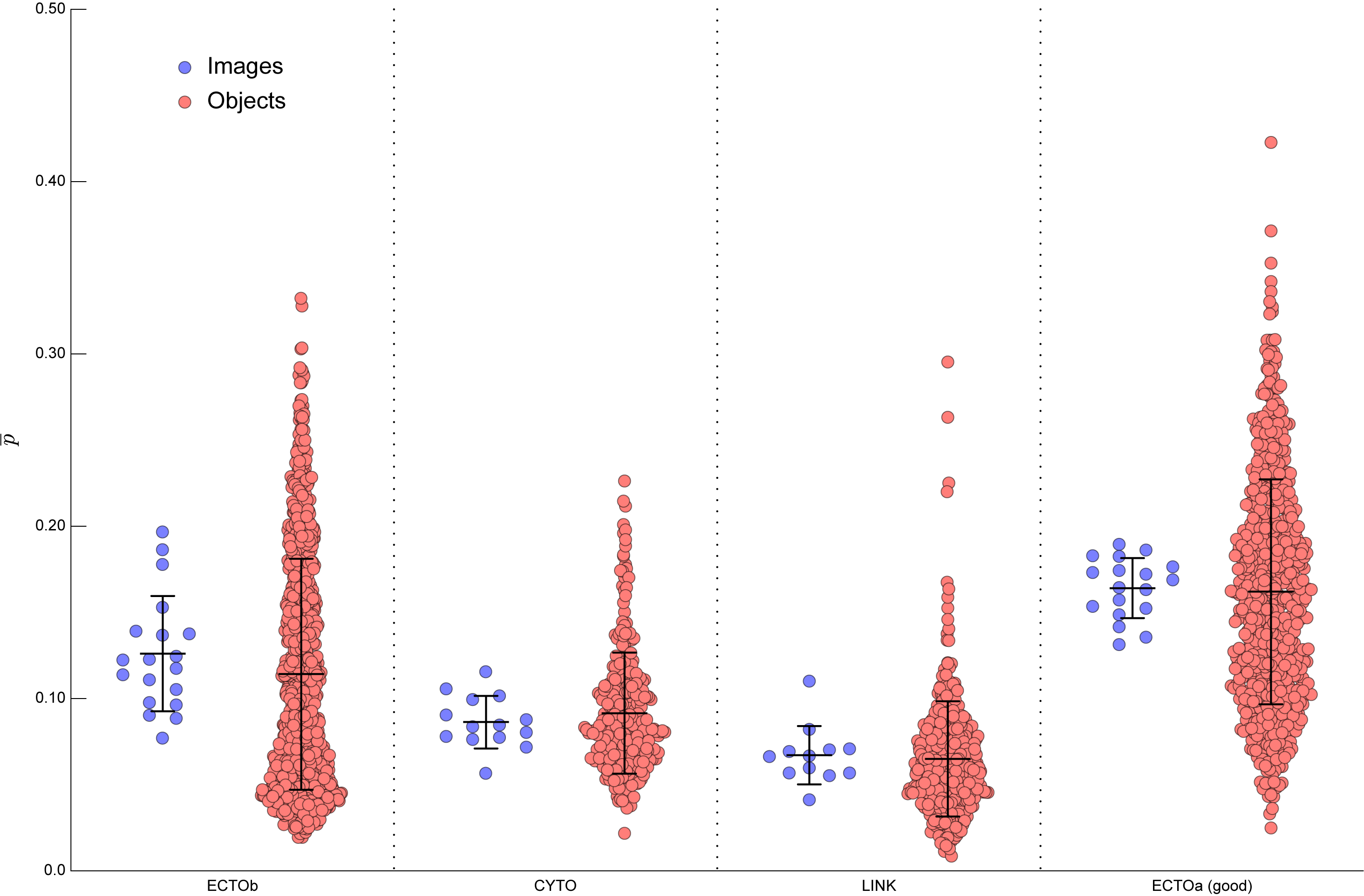

Supplement: S2 Fig — Swarmplots of mean order (p¯) for all of the images and the objects they contain for the ECTOb, CYTO, LINK, and ECTOa (good) datasets. Values correspond either to the average order among all masked pixels in an image (blue) or the average order among all of the pixels defining each object (red). (TIF) [file pcbi.1011723.s002.tif]
