# Supplementary material for: OOPS: Object-Oriented Polarization Software for analysis of fluorescence polarization microscopy images
Source: PLoS Comput Biol. 2024 Aug 12;20(8):e1011723. doi: 10.1371/journal.pcbi.1011723 (PMC11341096; doi:10.1371/journal.pcbi.1011723)
Supplement: S1 Text — (PDF) [file pcbi.1011723.s004.pdf]

Supplementary methods

Image types and visualization options

In addition to the raw order, azimuth, intensity, and mask images, OOPS produces a variety of additional outputs that combine multiple statistics into a single image. For example, the order-intensity overlay is constructed by merging the order and intensity images, with the intensity image acting as opacity mask (S1 Fig, *A*). This method is effective at dampening the noisy background regions of the order image, while not obscuring the sample itself. The contrast of the intensity and order channels can be set independently by the user, and the image can be colorized using a large selection of lookup tables (LUTs). The azimuth data are inherently more difficult to display in image form, due to the angular nature of the measurements. By default, the azimuth data are wrapped to the range [0°,180°] and displayed with a circular LUT where values of 0° and 180° map to the first and last colors in the LUT, respectively (S1 Fig, *B*). Similar to the order image, the azimuth image can be displayed using the intensity image as an opacity mask (*not shown*), which offers the same benefits mentioned above.

The final two image types combine all three measurements—intensity, order, and azimuth—into a single visualization. For the azimuth-order-intensity HSV, data are converted into an HSV image with hue, saturation, and value (brightness) determined by the azimuth, order, and intensity, respectively (S1 Fig, *B*). In this way, both the order and orientation information are represented in a single image, with the brighter, more ordered regions appearing more prominent. Alternatively, users can display azimuths as sticks overlaid upon the intensity image, where the orientation and length of each stick correspond to the azimuth and order of its corresponding pixel (S1 Fig, *C*). The length, width, color, density, and transparency of the sticks can all be customized by the user. In this case, the colors of each stick correspond to the azimuth in each pixel. Both representations show that individual pixel azimuths are preferentially oriented parallel to the long axes of the filaments, as expected.

Segmentation of punctate structures

Punctate structures were segmented using the “Puncta” scheme. First, the flat-field corrected FPM stack was averaged across all excitation polarizations and then normalized to the maximum intensity. To enhance punctate structures and correct for nonuniform illumination, a top-hat transform was performed using a disk-shaped structuring element with a radius of 3 pixels. The image was then median-filtered and again normalized to the maximum intensity. The enhanced image was binarized using an intensity threshold determined automatically with Otsu’s method. The resulting binary mask was used to segment the image into objects, which were defined as individual connected components with 4-pixel connectivity. After segmentation with the “Puncta” scheme, users can adjust the automatically selected threshold, though that was not done in this study.

Segmentation of filamentous structures
Filamentous structures were segmented using the built-in “Filaments” scheme. First, the flat-field corrected FPM stack was averaged across all excitation polarizations and then normalized to the maximum intensity. Filamentous structures were enhanced by applying a Hessian-based multiscale Frangi vesselness filter—using the MATLAB function, “fibermetric”—with a “thickness” of four pixels and a “structure sensitivity” equal to half of the maximum of the Frobenius norm of the Hessian. Elongated, linear structures were further enhanced by taking the pixelwise maximum of a series of morphological openings performed with a line-shaped structuring element with a length of 40 pixels rotating in 1° increments. The image was filtered with a Laplacian of Gaussian filter and an edge mask was generated from all zero-crossing locations in the filtered image. Holes in the edge mask were filled, and any H-connected or nearly H-connected pixels were removed. Next, any objects with eccentricity less than or equal to 0.5 or circularity greater than or equal to 0.5 were removed. Finally, any border pixels with a diagonal 8-connection were removed to produce the final mask. To label individual branches in the filament mask, the mask was first skeletonized and branchpoints of the skeleton were removed. Individual 8-connected branch skeletons were labeled, and the remainder of each branch was labeled using nearest neighbor interpolation.

Midline detection and midline tangent calculation
To estimate the positions of object midlines, individual object subimages were first extracted from the full mask image. The 8-connected boundary coordinates of each object were found using the MATLAB function, “bwboundaries”. Boundaries were smoothed using a Savitzky-Golay filter and then linearly interpolated such that the distance between each pair of neighboring points was identical. Smoothed, interpolated boundary points were then used as seed locations for a Voronoi tessellation and Delaunay triangulation. Edges of the Delaunay triangles were used to identify each pair of Voronoi polygons with a shared, finite edge. Voronoi edges were only retained if both vertices fell within the area of the smoothed object boundary. The remaining set of edges was converted into a weighted, undirected graph, with edge weights set by the Euclidean distance between each set of vertices. Next, the shortest path between each pair of nodes was calculated, with the maximum of those used to define the path between “endpoint” nodes. The nodes along the path between endpoint nodes were extracted into a separate subgraph and reordered to form a continuous edge path from endpoint to endpoint. Finally, the graph was converted back into a set of points, smoothed with a Savitzky-Golay filter, and linearly interpolated such that neighboring points were equally spaced.

The local orientation of the midline was estimated by calculating the tangent at each point using the finite difference method. For each set of midline points, M_i, with coordinates (x_i, y_i) and their neighboring points M_{i-1} and M_{i+1}:

$$\text{Tangent at } M_i = \frac{y_{i+1} - y_{i-1}}{x_{i+1} - x_{i-1}}$$

Midline tangents were wrapped to the range $[-\pi/2, \pi/2]$ and each object pixel was assigned the value of the tangent of the nearest midline point.

Azimuth statistics

Because azimuth values have π phase ambiguity, they were first transformed into complex unit vectors in the two-dimensional plane and mapped to a scale with a period of 2π prior to performing any calculations. After calculations were performed, unwrapped azimuths were returned to their original scale.

For a vector of N azimuths measured with respect to the horizontal direction in the image, $\alpha_{\text{image},n}$, we denote $\boldsymbol{\alpha}_{\textbf{image}} = (\alpha_{\text{image},1}, \dots, \alpha_{\text{image},n})$. The mean axial direction is:

$$\overline{\alpha}_{\text{image}} = \arg\left(\frac{1}{N}\sum_{n=1}^{N} \exp(2i\alpha_{\text{image},n})\right)/2$$

where N is the number of pixels in the object and i is the imaginary unit. For the purposes of calculating the angle of each azimuth with respect to the object midline, the object midline tangents, m_n, also have π phase ambiguity and are transformed identically to the azimuths. Thus, the average axial direction of object azimuths with respect to the object midline is given by:

$$\overline{\alpha}_{\text{midline}} = \arg\left(\frac{1}{N}\sum_{n=1}^{N} \exp\left(2i(\alpha_{\text{image},n} - m_n)\right)\right)/2$$

To calculate the azimuth circular standard deviation (s_0), we first calculated the mean resultant vector, which has the form:

$$\overline{r} = \frac{1}{N}\sum_{n=1}^{N} \exp(2i\alpha_{\text{image},n})$$

The resultant vector length was then calculated by taking the norm of the mean resultant vector.

$$R = \|\overline{r}\|$$

Finally, the resultant vector length was used to calculate the circular standard deviation of the azimuths, and the data were unwrapped and mapped back to their original scale.

$$s_0 = \frac{\sqrt{-2\ln R}}{2}$$

Description of test datasets
All of the data analyzed herein are included along with the software in the `sample_data` directory. In total, there are three datasets: **(Dataset 1)** Five groups of FPM images of A-431 cells expressing various Dsc2-mEGFP chimeras, located in `sample_data/Puncta/*/RAW`, where `*` is the name of the construct expressed and/or experimental condition; **(Dataset 2)** one group of FPM images of Cos-7 cells stained with Alexa Fluor 488 (AF488) phalloidin, located in `sample_data/Filaments/Cos-7_F-Actin/RAW`; and **(Dataset 3)** two groups of FPM images of human umbilical vein endothelial cells (HUVECs) grown in static conditions or under fluidic shear stress (FSS), located in `sample_data/Filaments/HUVEC_F-Actin/*/RAW`, where `*` is either `Static` or `Flow`.

For each group of images, the `RAW` directory contains both the raw FPM stacks as well as three flat-field correction (FFC) stacks (`FFC_*.nd2`). Additionally, one level above each `RAW` directory is a plain text file, `info.txt`, which gives a short summary of the sample characteristics, treatment conditions (if applicable), and acquisition settings.

Processing Dataset 1
To replicate the analyses in this study for **Dataset 1** (Fig 2), follow the instructions in the OOPS User Manual to load, process, and visualize the data in `sample_data/Puncta`. When first starting a new project, create five groups—one for each group of images in the dataset—named according to each of the folders in `sample_data/Puncta`. At the segmentation step, use the "Puncta" scheme. Do not adjust any intensity thresholds or delete any objects manually. After the initial processing steps are complete, sort the objects into low and high S/B groups using k-means clustering. To achieve the exact clustering results shown in this study, select "Local S/B" as the only variable, "auto" for the "k selection mode", "CalinskiHarabasz" for the "Criterion", "cosine" for the "Distance metric", and "z-score" for the "Normalization method". Run k-means clustering as described in **subsection 10.2** of the OOPS User Manual. Note that these parameters were chosen for demonstration purposes to highlight the relationship between local S/B and order. The clustering solution may vary significantly with different parameters. For example, selecting the "Silhouette" criterion combined with the "cityblock" distance metric produces very similar results, while selecting the "Silhouette" criterion with the "sqeuclidean" distance metric produces drastically different results. The choice of parameters should be chosen carefully based on the desired downstream analyses. After completing the analysis, the plots in Fig 2D, 2E, 2G, and 2H can be reproduced by adjusting the Scatterplot and Swarmplot appearance settings, as described in **subsection 8.1** and **subsection 8.3** of the OOPS User Manual, respectively. For the visualizations shown in Fig 3, switch to the group corresponding to the data in `sample_data/Puncta/Dsc2-ECTOb/RAW`. To reproduce the azimuth stick overlays shown in Fig 3B and 3D, locate **Object 37** in the image, `Dsc2-ECTOb_095`, and use the object viewer as described in **Section 7** (S1 Fig). To reproduce the plots in Fig 3E and 3F, view and adjust the polar histograms as described in **subsection 8.4**. Set the "Variable" to "Mean Azimuth" for Fig 3E and "Mean Azimuth (Midline)" for Fig 3F.
Processing Dataset 2

For **Dataset 2**, follow the instructions in the OOPS User Manual to load, process, and visualize the data in `sample_data/Filaments/Cos-7_F-Actin/RAW`. No object-based analyses of **Dataset 2** were included in this study, so the segmentation step is optional. Additionally, while the dataset contains many images, the only image shown is `Cos-7_F-Actin_40.nd2`. After computing the FPM statistics, reproduce the image in S1 Fig *A* by selecting the "order-intensity overlay", as described in **step 9** of **subsection 6.2** in the OOPS User Manual. Select "jet2" as the colormap and set the display limits of the average intensity and order images to match those shown in the figure. See **subsection 6.1** and **subsection 6.2** of the manual for details on viewing and adjusting the appearance of the "intensity" and "order" images, respectively. Reproduce the image in S1 Fig *B* by selecting the "Azimuth-Order-Intensity HSV overlay", as described in **step 7** of **subsection 6.3**. To reproduce the magnified images shown in S1 Fig *C*, use the pan/zoom tool to zoom into the regions of interest shown in S1 Fig *A* and *B*, as described in **step 7** of **subsection 6.1**. While zoomed in to a region of interest, reproduce the azimuth stick overlay images by adjusting the appearance of the azimuth sticks, as described in **step 9** of **subsection 6.3**.

Processing Dataset 3

For **Dataset 3** (Fig 4 and Fig 5), follow the instructions in the OOPS User Manual to load, process, and visualize the data in `sample_data/Filaments/HUVEC_F-Actin`. When first starting a new project, create two groups—one for each group of images in the dataset—named "Flow" and "Static". At the segmentation step, use the "Filaments" scheme. After the initial processing steps are complete, manually sort the objects into two groups ("Long" and "Short") based on "Midline Length" as described in **subsection 9.2**. To achieve the exact results shown in this study, any filaments for which the midline could not be detected must be removed first. First, create two new labels named "Long" and "Short". Select all objects with "Midline Length" > 0 and assign them the "Long" label. Any objects remaining in the "Default" label do not have a successfully detected midline; delete the "Default" label and its objects. Next, clear the object selection, select all objects with "Midline Length" < 161.6 pixels (corresponding to 17.5 μm at a pixel size of 0.1083 μm/pixel), and assign the selected objects the "Short" label. At this point, the "Short" label should hold all filaments shorter than 17.5 μm, while the "Long" label holds filaments with lengths greater than or equal to 17.5 μm. To reproduce the images in Fig 4A, first switch to the "Static" group and select the image, `HUVEC_F-Actin_Static_015`. To view the "mask", switch to the "Mask" view as described in **step 1** of **subsection 5.1**. To view and adjust the "intensity" and "azimuth HSV" images, use the same strategy described for **Dataset 2**. Repeat these steps on the image, `HUVEC_F-Actin_FSS_015`, in the "Flow" group to reproduce the images in Fig 4B. To reproduce the polar histograms in Fig 4C and 4D, use the same strategy described for **Dataset 1** for both the "Flow" and "Static" groups. To reproduce the plots in Fig 5, refer to **subsection 8.1–8.3** of the OOPS User Manual. To reproduce the exact scatter plot matrix shown in Fig 5C, select the following "Variables": "Mean Azimuth", "Azimuth Circular Standard Deviation", "Mean Azimuth (Midline)", "Midline Length", and "Mean Order"; set the "Color mode" to "Label"; and set the "Diagonal display" to "Grouped histograms".
